# Complete Eversion of the Uterus in a Mare, Two Cases of Fractured Jaw

**Published:** 1899-06

**Authors:** Daniel D. Lee

**Affiliations:** Boston, Mass.


### COMPLETE EVERSION OF UTERUS IN A MARE—TWO CASES OF FRACTURED JAW.

By Daniel D. Lee, M.D.V.,
BOSTON, MASS.

A mare, used for saddle, came out of the arena at half-past five, and immediately after the water-bag showed, and a dead colt, about ten months along, was born.

For an hour or two all went well; then the mare became slightly uneasy. At eleven o'clock she was resting quietly, and, leaving a dose of soothing medicine, I left her for the night.

At 3.30 A.M. she threw out the uterus and part of horns; became frantic and bled freely. I was sent for, and by using side lines succeeded in returning the uterus. I had with me the rubber bag and bulb of a thermocautery, which I placed in the womb after tying the rubber tube, and then blew up the bag; this prevented the accident recurring. Through the day she did well, and did not strain badly. I had a man with her every minute.

In the afternoon I took over a tracheotomy tube, but found her so well that I did not put it in. About ten o'clock that night she strained very badly, and at 11.30 the tube was put in the trachea; this at once stopped the straining, and I removed the bag from the uterus.

I had not allowed her to lie down, and as she was hard to keep on her feet I slung her. No other unfavorable symptoms appeared, and on the third day I took out the tube. On the fifth day I took her out of slings, and in ten days she was well. The owner had no idea that the mare was with foal, and if she had stayed in the arena five minutes longer than she did a large class of riders, in which there were many ladies, would have been treated to a surprise.

The mare passed an examination as to soundness six weeks before the accident.

*Two Cases of Fractured Jaw.*

A large bay team horse caught his upper teeth on the spreader and disarticulated the right premaxillary bone with his three incisor teeth. The bone and teeth projected outward into the lip, and were immovable. I drove the bone and teeth back with a hammer, and used nothing to secure them. The horse never lost a meal, and made a perfect recovery except that the three incisor teeth on that side dropped one-eighth of an inch below the others.

A big chestnut driving horse ran away and broke the lower jaw on the right side, so that the right nipper tooth was entirely exposed and could be taken out with the fingers. The fracture was not entirely through the symphysis, but a piece about six inches long was wedged outward into the lower lip, this piece carrying the divider and corner tooth. The injury was thirty-six hours old, and the horse had been condemned as incurable. I drew the jaw together, using an ordinary twister and turning it up until the jaw was in place. The divider tooth was broken and had to be partly removed. The jaw was not secured in any way, and the horse fed without trouble. He was discharged, convalescent and able to have a bit in his mouth, in ten days.

